# Change in serum level of vitamin D and associated factors at early phase of bone healing among fractured adult patients at University of Gondar teaching hospital, Northwest Ethiopia: a prospective follow up study

**DOI:** 10.1186/s12937-017-0277-y

**Published:** 2017-09-05

**Authors:** Yalelet Fentaw, Haile Woldie, Solomon Mekonnen, Adino Tesfahun Tsegaye

**Affiliations:** 1 Department of Nutrition, University of Gondar Teaching Hospital, Gondar, Ethiopia; 2 Department of Human Nutrition, Institute of Public Health, College of Medicine and Health Sciences, University of Gondar, Gondar, Ethiopia; 3 Department of Epidemiology and Biostatistics, Institute of Public Health, College of Medicine and Health Sciences, University of Gondar, Gondar, Ethiopia

**Keywords:** Serum level of vitamin–D, Fractured bone, Inadequate milk intake, Dietary diversity score, Northwest Ethiopia

## Abstract

**Introduction:**

Currently, Vitamin D deficiency is a major public health problem and it affects more than one billion people worldwide. Vitamin D is crucial for bone mineralization and ossification. Patients with fractures need Vitamin D for the healing of their fractured bone. The current study was carried out to determine if there is change in the serum level of Vitamin–D associated with factors at early phase of fractured bone healing (ossification) process among adult fractured patients at University of Gondar teaching hospital, Northwest Ethiopia.

**Methods:**

This facility-based prospective follow up study was conducted from March to June 2016. Data was collected by an interviewer, and pretested and structured questionnaires were used. Biological samples were collected to determine the serum level of vitamin–D in all subjects. In addition, X–Ray findings were used to determine the early phase of bone healing process. Data was entered into EPI INFO version 3.5.3 and analyzed using the Statistical Package for Social Sciences (SPSS) version 20. Both bivariate and multivariate logistic regression analysis was done to screen for factors associated with decreased serum levels of Vitamin–D. In the Multivariate regression analysis, those variables which had a P–value of <0.05 were considered as independently associated with change in serum level of Vitamin–D.

**Results:**

A total of 118 adult patients with fractures participated in this study. The prevalence of patients’ with decreased serum levels of vitamin–D at post-test was 63.6% [95% CI; (0.551–0.720)]. Inadequate intake of milk and milk products in the 1st week of fracture [AOR = 95%CI: 0.20 (0.05–0.90)], Poor Dietary Diversity Score [AOR = 95% CI: 29.1 (2.27–371.65)], and ossified bone [AOR =95% CI: 4.10 (1.12–14.95)] showed statistically significant association with decreased serum level of Vitamin–D.

**Conclusion and recommendations:**

Decreased serum level of Vitamin–D at early phase of fractured bone healing process was found in the majority of patients (>63%) raising concern for Vitamin D deficiency to be a significant public health problem in the study population. It was statistically associated with: poor dietary diversity score, in adequate intake of milk and milk products in the 1^st^one week of fracture and ossified (healed) bone. Introducing hospital based Vitamin–D supplementation and integrated with health and nutritional education is a vital intervention needed to improve serum levels of Vitamin–D.

## Background

Vitamin–D is an essential micronutrient for the maintenance of bone quality and healing. It is actively involved in bone formation, mineralization, and maintenance of neuromuscular function [1]. It also regulates bone metabolism through activation of the Vitamin–D receptors found in osteoblasts [2]. Vitamin–D has a primary physiological role in maintaining extracellular calcium ion levels in the human body [3, 4].

Globally, Vitamin–D deficiency is a public health problem and it affects more than one billion people [5]. In developing nations, the magnitude of Vitamin–D deficiency ranges between 30 and 90% [6–8] and it is higher in older age and female gender [7]. Vitamin–D deficiency results from inadequate synthesis in the skin decreased dietary intake or impaired vitamin–D activation in the liver and kidney [9]. Low serum Vitamin–D levels of (25 [OH] D) in adults can precipitate or exacerbate osteopenia, osteoporosis, and cause osteomalacia and muscle weakness [10, 11]. It also causes impaired calcium absorption which can lead to growth retardation; skeletal abnormalities; and increases the risks of bone fractures [12, 13]. In its’ severe form, Vitamin–D deficiency leads to secondary hyperparathyroidism, bone loss, muscle weakness, and causes increased risk of many infections and chronic diseases (cancers and cardiovascular diseases) [14–16].

The cause of low serum levels of Vitamin–D is multi-factorial [17, 18]. Different settings around the world have identified multiple risk factors affecting levels of vitamin–D. For example; age and sex [18–21], socio-economic status [22–24], nutritional status (obese individual) [25], history of chronic and acute illness, repeated history of bone fracture [26], dietary diversity, clothing style, skin color [27], ethnicity, latitude (lower latitude), cultural practice [28], degree of physical activity (limiting outdoor) [27], geographical location and season (winter) [29] were significantly associated with decreased serum level of Vitamin–D.

Understanding the effect of low Vitamin D levels, and related risk factors, on the management of patients’ with fracture is critical. However, there is scarce knowledge of the effect of decreased serum levels of Vitamin–D during early phase of fractured bone healing process among patients with fractures in Ethiopia, particularly in the Northwest part of the country. Therefore, this study aimed to assess changes in serum level of Vitamin–D and its predictors at early phase of bone healing process among newly admitted fractured adult patients at University of Gondar teaching hospital, Northwest Ethiopia.

## Method and materials

### Study design and setting

This institutional based prospective follow up study was conducted from March–June 2015. The study was undertaken at Amhara regional state, University of Gondar teaching hospital. The hospital is located in Gondar, Northwest Ethiopia. It is located 738 km far from the capital city of Ethiopia, Addis Ababa at an elevation of 2215 m. The University of Gondar teaching hospital serves more than five million people. The services are predominantly delivered through the four major wards of Surgery, Internal medicine, Gynecology and Obstetrics, and Pediatrics with other sub specialty and minor units. Currently, the teaching hospital provides service for more than 20,000 surgical cases per year, among them are on average1450 patients with fractures annually. The majority of these patients are treated in different departments of the hospital like the emergency room, orthopedics, surgery, recovery, and the minor trauma unit.

### Sample size determination, sampling procedure and study subjects

The required sample size for the study was determined using a single population proportion (P) formula with the following assumptions; the prevalence of change of serum level of Vitamin–Dwas taken as (P) = 50% and with the consideration of the following assumptions; a 95% confidence level, and 5% margin of error (d). At last, a minimum sample size of 118 was calculated after anticipating a 15% non–response rate. Systematic random sampling technique was employed to recruit study participants. The sampling interval was calculated by considering the average patient flow after reviewing surgical case registration book of the teaching hospital, for 2015. After determination of the sampling interval as 3.2, every 3rd fractured patient was included in the study. All patients aged ≥18 years with new traumatic bone fracture who visited the teaching hospital within 48 h after injury could participate in the study. Those patients with finger and non-traumatic fractures were not included in this study.

### Data collection instrument and procedures

An interviewer administered, pretested and structured questionnaire was employed to collect the required information for this study. The questionnaire included socio-demographic and economic characteristics, health and dietary habits and other related characteristics. To check its consistency, the questionnaires were first prepared in English then translated to the local language (Amharic) and back to English by professional translators. A total of four health professionals (two Nurses as data collector, one laboratory technologist for biological sample analysis, and one Public Health Officer as supervisor) were recruited for the data collection process.

Biological samples to measure the serum level of 25hydroxy Vitamin–D (25–OHD) was collected within the first 48 h of fracture. Strict aseptic technique and a separate lancet for each study participants were used. A 5 ml of venous blood was carefully collected from the left hands of fractured patients. The serum was immediately frozen and stored between 2 °C and 8 °C until the laboratory test was conducted within 5 days. Serum concentration of 25-hydroxy Vitamin D (25–OH–D) status was defined by the mini Vitek Immune Diagnostic Assay System (VIDAS) machine. The change in serum level of Vitamin–D was classified into two categories as decreased and increased by considering 1 month’s follow up result from the baseline measurement. Decreased serum level of Vitamin–D was defined as a 25–OHD level of less than 50 nmol/L (20 ng/ml) [30].

X–ray (Radiography) findings were used to determine the site and healing process of fractured bone. Early phase of bone healing process was measured in the first 1 month of fracture. The healing process was defined as: healed bone, when the radiologist determined the fractured bone is ossified/calcified after 1 month of visit.

Nutritional status of the patient was assessed by taking anthropometric body measurement of height and weight. Based on the severity of fracture and type, two techniques were employed to take the height measurement of study subjects. Among a total of 118 patients; 73 were measured in a standing position with adult scale astormeter to the nearest 0.1 cm. The remaining 45 patients’ heights were measured in a recumbent position to the nearest 0.1 cm by using a board with an upright wooden base and a movable headpiece, on a flat surface. Weight of the patient was taken by an adult scale astor electronic weight scale with the calibration of 100 g unit. The scale was adjusted to zero before weighing every fractured patient. All study participants were without any shoes during the weight and height measurements.

A mathematical calculation of Body Mass Index (BMI) was used to determine the nutritional status of study subjects. Nutritional status was defined as poor nutritional status (when the BMI level of <18.5 kg/m^2^), good nutritional status (when BMI level was 18.5–24.9), Overweight (when BMI of 25–29.9), Obese (when BMI of ≥30) [31].

Dietary diversity score was determined by using a 24 h recall method. The patients were requested to list what they consumed in the previous 24 h of the survey. Dietary diversity score was then computed based on 9 food groups as recommended by FAO which are comprised of: grains, roots and tubers; legumes and nuts; dairy products; flesh foods (meat, fish, poultry and organ meats); eggs; vitamin A rich fruits and vegetables; other fruits and vegetables. Finally, dietary diversity scores (DDSs) were calculated; as poor dietary diversity score, (when the patient consumed ≤3 food groups), medium dietary diversity score (when the patient consumed 4–5 food groups) and high dietary diversity (when the patient consumed ≥6 food groups) in the previous 24 h of the survey [32].

The living standard of the household was computed using a composite indicator for urban and rural residents. The asset information was determined via a principal components analysis. The index was constructed using the selected household asset information for urban and for rural residents. The household wealth index was categorized into three categories (Poor, Middle and Rich) [33].

### Data quality control

In order to ensure data quality, questionnaires were translated by professional translators. In addition, a 2 day training was given to recruited data collectors and supervisor. The training was focused on the objective of the study, technique of interview, how to collect the samples, and relevant ethical issues (respondent’s right, confidentiality of information etc.). Three days prior to the actual data collection, the data collection tool was pretested among 5% of study subjects out of the study area. During the pretest, the applicability of the procedures and tools were evaluated. ll questioners were checked for completeness, clarity and consistency by the supervisor. Furthermore, the investigators coordinated the overall activities of data collection process throughout the present study.

### Data processing and analysis

Data were entered into EPI INFO version 3.5.3 and analyzed using the Statistical Package for Social Sciences (SPSS) version 20. Before analysis the data was cleaned thoroughly to check for errors during entry**.** Descriptive statistics, including frequencies and proportions were used to summarize the study variables. A binary logistic regression was used to investigate factors associated with decreased serum level of vitamin–D. Those predictor variables with a P–value of <0.2 in the bivariate analysis were exported to multivariate analysis to control the possible effect of confounders. The Adjusted Odds Ratio (AOR) at a 95% confidence interval and P–value of ≤0.05 was used to declare the strength of association with decreased serum level of Vitamin–D.

## Results

### Socio–demographic and clinical characteristics of the study participants

A total of 118 (103 male and 18 female) participants with a 100% of response rate were included in the study. The mean age of the study subjects were 36.87 year with a standard deviation (SD) of ±13.4 years. More than half (62.7%) of study subjects were rural dwellers. Orthodox religion accounted for 89.0% study participants. Seventy two (61.0%) subjects were farmers and 80 (67.8%) were married. The family size of the study subjects ranged from 1 to 5 with mean family size of 4.1 (Table 1).

**Table 1:** Socio-economic and demographic characteristics of fractured adult patients at University of Gondar teaching hospital, Northwest Ethiopia, 2016 (*n* = 118)

Background characteristics	Frequency(Percent)
Place of residence	
Urban	44(37.3)
Rural	74(62.7)
Age (in Years) of the respondent	
18–30	54(45.8)
31–40	25(21.2)
41–50	20(16.9)
50–71	19(16.1)
Sex of the respondent	
Male	103(87.3)
Female	15(12.7)
Religion of the respondent	
Orthodox	105(89.0)
Muslim	13(11.0)
Ethnicity of the respondent	
Amhara	113(95.8)
Tigre	5(4.2)
Marital Status of the respondent	
Married	80(67.8)
Single	34(28.8)
Widowed	4(3.4)
Educational Status the respondent	
Not read and write	27(22.9)
Read and write	31(26.3)
Elementary school(Grade 1–4)	13(11.0)
Junior high school(Grade 5–8)	25(21.2)
Senior high school(Grade 9–10)	16(13.6)
Preparatory(Grade 11–12)	4(3.4)
Higher education (> Grade 12)	2(1.7)
Occupation of the respondent	
House wife/work	10(8.5)
Merchant	19(16.1)
Daily Labourer	11(9.3)
Governmental employed	6(5.1)
Farmer	72(61.0)
Family size of the respondent	
1–4	91(77.1)
≥ 5	27(22.9)
Household Wealth Index	
Poor	38(32.2)
Medium	41(34.7)
Rich	39(33.1)

### Dietary intake and dietary diversity scores related characteristics

Teff, Maize and Sorghum were the three most common staple diets in the study area. Of 98.3% participants, The frequency of meals was <3 in 98.3% of participants prior to the first visit. More than 50 and 47.5% study participants reported that they had less than three meals per day and four meals per day, respectively, a month after the fracture. (Table 2).

**Table 2:** Dietary intake and Dietary Diversity Scores related characteristics among fractured adult patients at University of Gondar teaching hospital, Northwest Ethiopia, 2016 (*n* = 118)

Characteristics	Frequency (Percent)
Consumption of milk and milk product at 1st visit	
Yes	37(31.4)
No	81(68.6)
Milk and milk products after a month of fracture	
Yes	68(57.6)
No	50(42.4)
Meal frequency per a day of 1st visit	
≤ 3 Times	116(98.3)
≥ 4 Times	2(1.7)
Meal frequency per day of after a month of fracture	
≤ 3 Times	62(52.5)
≥ 4 Times	56(47.5)
Fruit and vegetable consumption per week of after fracture	
Yes	31(26.3)
No	87(73.7)
Staple diets of the family (*n* = 223)	
Teff	92(41.3)
Maize	70(31.4)
Sorghum	61(27.3)
Household source of food	
Own production	54(45.8)
Purchase	42(35.6)
Both growth & purchase	22(18.6)
Availability of home grading	
Yes	19(10.6)
No	99(89.4)
Types of foods cultivated in home garden (*n* = 9)	
Fruits and vegetables	17(77.8)
Others	2(22.2)
DDSs in previous 24 h of 1st visit	
Poor	74(62.7)
Medium	39(33.1)
High	5(4.2)
DDSs in previous 24 h after a month of fracture	
Poor	29(24.6)
Medium	62(52.5)
High	27(22.9)

**Fig. 1:**
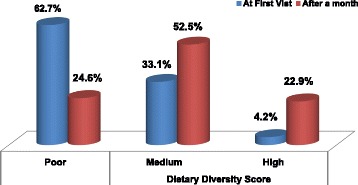
Dietary diversity scores at baseline and after one month among adult fractured patients in University of Gondar Teaching Hospital, Northwest Ethiopia 2016

A 24 h dietary recall technique was employed to determine the dietary diversity scores in the previous 24 h of the interview. During the time of first visit, nearly 60 % of study participants received poor dietary diversity scores. In contrast, the proportion of individuals with low dietary diversity scores was 24.6% 1 month after the fracture. (Fig. 1).

### Co–morbidity and nutritional status related characteristics

Ten (8.5%) of the respondents had a history of a known chronic disease and were currently on medications during the study. Among them, three had both diabetes mellitus and hypertension, four had only hypertension, two had chronic asthma, and one had tuberculosis. Almost all patients with a chronic disease history received health and nutrition education for the dietary management of their chronic diseases.

Based on the mathematical calculation of body mass index (BMI), 89.0% study subjects had normal nutritional status in this study (Table 3).

**Table 3:** Co–morbidity and nutritional status related characteristics among fractured adult patients at University of Gondar teaching hospital, Northwest Ethiopia, 2016 (*n* = 118)

Characteristics	Frequency (Percent)
History of any known chronic disease	
Yes	10(8.5)
No	108(91.5)
Nutrition education for the dietary management of the diseases	
Yes	9(90.0)
No	1(10.0)
Nutritional status by body mass index (BMI)	
Normal	105(89.0)
Under–Weight	9(7.6)
Over–Weight	4(3.4)

### Change in serum level of vitamin D

Nearly 70 % of study participants (69.49%) enrolled were treated as an inpatient and the rest as outpatients. There was a difference between the pre-tests and post-tests done at baseline and after 1 month (P–value 0.053) after the fracture. Serum levels of Vitamin D significantly decreased during the first month following a fracture in the majority of the study community [63.6%: 95% CI; (0.551–0.720)] compared to the base line.

### Predictors of change in serum level of vitamin–D

In the bivariate logistic regression analysis age, place of residence, time of accident, milk consumption at first visit, religion, Dietary Diversity Scores (DDSs) at 1st visit, healed bone (ossification), Dietary Diversity Scores (DDSs) in the 1st week of a month and history of milk and milk products consumption in the 1st week of a month were significantly associated with decreased serum level of Vitamin–D. In the multivariable regression analysis, bone healing (ossified bone), milk and milk product consumption in the 1st week of a month and dietary diversity score in the 1st week of a month were listed out as strong predictor of decreased serum levels of Vitamin–D. But having chronic co morbidity has no any statistically significant association with change in serum level of Vitamin D. Since the *P*-value was > 0.2 in the bivarable model, it was not included in the multi-variable model. Accordingly, the likelihood of decreased serum levels of Vitamin–D was more than four times higher among patients who had ossification of the fractured bone than those who had not [AOR = 95%CI: 4.1 (1.12–14.9)]. The odds of decreased serum level of Vitamin–D was greater than 29 times among patients who had poor dietary diversity scores in 1st week than those who had high DDSs[(AOR = 95%CI: 29.05 (2.27, 371.65)]. Likewise, the odds of decreased serum level of Vitamin–D during early phase of bone healing process was 80% more likely among patients who didn’t consumed milk and milk products in the 1st week after fracture than those who had[AOR = 0.20; 95% CI (0.05, 0.90)] (Table 4).

**Table 4:** Factors associated with change in serum level of Vitamin–D among fractured adult patients at University of Gondar teaching hospital, Northwest Ethiopia; 2016 (*n* = 118)

	Change in serum level of Vitamin–D after a month			
Characteristics	Increased (#)	Decreased (#)	COR (95% CI)	AOR (95% CI)
Age (in Years)				
18–30	24	30	*1*	*1*
31–40	6	19	2.53 (0.87, 7.34)	3.08 (0.58, 16.32)
41–50	8	12	1.2 (0.42, 3.41)	1.27 (0.28, 5.78)
> 50	5	14	2.24 (0.71, 7.10)	1.27 (0.26, 6.18)
Residence				
Urban	21	23	2.16 (0.99, 4.68)	1.53 (0.46, 5.12)
Rural	22	52	*1*	*1*
Religion				
rthodox	36	69	2.24 (0.70, 7.15)	1.53 (0.22, 10.45)
Muslim	7	6	*1*	*1*
Time of accident				
1–12 h	32	65	*1*	*1*
≥ 13 h	11	10	2.23 (0.86, 5.81)	2.37 (0.51, 11.09)
Consumption of milk and milk products at 1st visit				
Yes	9	28	*1*	*1*
No	34	47	2.25 (0.94, 5.38)	2.41 (0.55, 10.52)
DDSs at first visit				
Poor	14	60	6.43 (0.98,42.19)	0.55 (0.05, 6.57)
Medium	26	13	0.75 (0.11, 5.06)	0.24 (0.02, 2.57)
High	3	2	*1*	*1*
Fractured bone ossification				
Yes	30	13	11.01(4.55,26.63)	4.10(1.12,14.95)*
No	13	62	*1*	*1*
DDSs after a month of fracture				
Poor	1	28	98 (10.95,876.79)	29.1 (2.27,371.65)*
Medium	21	41	6.83 (2.39,19.50)	3.54 (0.95, 13.21)
High	21	6	*1*	*1*
Consumption of milk and milk products after a month of fracture				
Yes	40	28	*1*	*1*
No	3	47	0.05 (0.01, 0.16)	0.20 (0.05, 0.90)*

Note:*Significant association in multivariate analysis

*DDSs* dietary diversity scores, *COR* crude odds ratio, *AOR* adjusted odds ratio

## Discussion

This study was aimed to determine if there is significant change in serum levels of Vitamin–D in conjunction with associated risk factors that may adversely affect the early phase of bone healing following a fracture. The prevalence of decreased serum levels of vitamin–D was 63.6% [95% CI; (0.551–0.720)]. Determinants of change in serum level of vitamin D were: inadequate intake of milk and milk products in the 1st week following a fracture, poor Dietary Diversity Score, and ossified bone.

Vitamin–D is essential micronutrient for bone healing and it is actively involved in bone formation and mineralization [1]. Since patients consumed more vitamins –D for the healing of their bone, the serum level of vitamin D becomes decreased. The prevalence of decreased Vitamin D levels (63.6%) were much higher than studies from Italy [21.6%] [34], India [32.3%] [35], and Singapore [34.5%] [23]. Variation among results could be due to the difference in skin color of study participants. Unlike other studies, the participnts of this study are dark–skinned individuals and they require greater duration of sunlight exposure than those light–skinned counterparts to synthesize comparable amounts of Vitamin–D [36, 37]. The other possible reason might be due to difference in geographical location of the study participants. African people have greater risks of low serum Vitamin–D levels [38–42] and, Vitamin–D deficiency is a common contributor of bone fracture [11]. Additionally, due to reduced sunlight exposure of hospitalized and institutionalized individuals due to fracture/disability, they might have higher risk of Vitamin–D insufficiency [17, 19, 33], which may further contribute to high prevalence of Vitamin–D deficiency. Moreover, there may be Vitamin–D supplementation and micronutrient fortification programs in other countries. Vitamin D supplementation, nutritional health education and fortification programs may help improve the serum Vitamin–D level and increase the healing process of fractured bone.

Ossified bone (healed bone) was statistically associated with decreased serum levels of Vitamin–D. Decreased serum level of Vitamin–D was higher among individuals with ossified bone than non ossified bone 1 month after fr. This result was congruent with a study done in Singapore [23] and Russia [43]. This was evidenced by the fact that Vitamin–D is critical to the bone healing pathway of fractured bones. It is important in the formation and mineralization of the callus; it acts directly on osteoblasts, stimulates the synthesis of osteocalcin, while also acting on osteoclasts to stimulate bone resorption [29, 44–47]. Due to its role in the formation and mineralization of the callus, serum level of Vitamin–D decreases when fractured bone heals [24]. Furthermore, Vitamin–D stimulates chondrogenesis by cell proliferation and promotion of matrix protein synthesis, during the fractured bone healing process [48–50], which can result in decreased serum levels of Vitamin–D.

Dietary diversity score in the previous 24 h of the survey was independently associated with change (often decrease) in serum level of Vitamin–D. Poor DDSs in the 1st week following a fracture increased the odds of decreased serum level of Vitamin–D. The result was similar in a study in six regions of the world (Asia, Europe, Latin America, Africa, North America, and Oceania) [51]. The possible reasons could be; dietary diversity scores in the previous 24 h of an individual positively correlated with adequate micronutrient density of the diet [52–54], including Vitamin–D. In addition, consumption of undiversified foods may increases inadequate intake of Vitamin–D rich food sources, contributing to decreased serum levels of Vitamin–D.

Inadequate intake of milk and milk products 1 month after fracture was one of the important risk factors affecting change in serum level of Vitamin–D. The result was in agreement with results from six regions of the world (Asia, Europe, Latin America, Africa, North America, and Oceania) [51]. Since Vitamin–D as the primary regulator of calcium absorption, this may be an important contributing cause for low levels of vitamin D [12]. Vitamin D influences calcium levels primarily by controlling the absorption of calcium from the intestine, through direct effects on bone and parathyroid hormone (PTH) secretion [13]. Decreased serum level of Vitamin–D has been linked to the increased levels in alkaline phosphatase and parathyroid hormone and decreased calcium levels [55]. This leads to decreased calcium absorption and ultimately the release of calcium from the bones. Even milk and milk products which are rich dietary sources of calcium, when Vitamin D is below20 nmol/L, this results in extremely decreased intestinal calcium absorption in the body [56].

The results of this study may applicable to many populations; however, the main limitation of this study lied in the cross–sectional nature of the study. Fortunately, the study noted important trends that can be used to design health and nutrition interventions to improve serum level of Vitamin–D among adult patients with fractures in Ethiopia and possibly around the world. More research is needed to apply important finding more broadly.

In conclusion, decreased serum level of Vitamin–D during early phase of bone healing process was high in the study community. Poor dietary diversity score, bone healing (ossification) status and inadequate intake of milk and milk products after 1 month following a fracture was significantly associated with decreased serum levels of Vitamin–D. Introducing hospital based Vitamin–D supplementation in conjunction with health and nutritional education is a vital intervention to improve serum levels of Vitamin–D and promote bone healing in the early phase following a fracture in order to maximize health and healing (ossification) process in the study area.

## Abbreviations

25- OHD: 25- Hydroxyl Vitamin - D
AOR: Adjusted Odds Ratio
BMI: Body Mass Index
DDS: Dietary D﻿iversity Score
FAO: Food and Agriculture Organization
IPH: Institute of Public Health
PTH: Parathyroid Hormone
SPSS: Statistical Package for Social Science
VIDAS: Vitek Immune Diagnostic Assay System
